# Comparison of array-based comparative genomic hybridization with gene expression-based regional expression biases to identify genetic abnormalities in hepatocellular carcinoma

**DOI:** 10.1186/1471-2164-6-67

**Published:** 2005-05-09

**Authors:** Kyle A Furge, Karl J Dykema, Coral Ho, Xin Chen

**Affiliations:** 1 Bioinformatics Special Program, Van Andel Research Institute, 333 Bostwick Ave. NE, Grand Rapids, MI 49503, USA; 2 Dept of Pharmaceutical Sciences, University of California, S816 513 Parnassus Ave., San Francisco, CA 94143-0046, USA; 3 Cancer Center, University of California, S816 513 Parnassus Ave., San Francisco, CA 94143-0046, USA; 4 Liver Center, University of California, S816 513 Parnassus Ave., San Francisco, CA 94143-0046, USA

## Abstract

**Background:**

Regional expression biases (REBs) are genetic intervals where gene expression is coordinately changed. For example, if a region of the genome is amplified, often the majority of genes that map within the amplified region show increased expression when compared to genes located in cytogenetically normal regions. As such, REBs have the potential to act as surrogates for cytogenetic data traditionally obtained using molecular technologies such as comparative genomic hybridization. However as REBs are identified using transcriptional information, detection of REBs may also identify local transcriptional abnormalities produced by both genetic and epigenetic mechanisms.

**Results:**

REBs were identified from a set of hepatocellular carcinoma (HCC) gene expression profiles using a multiple span moving binomial test and compared to genetic abnormalities identified using array-based comparative genomic hybridization (aCGH). In the majority of cases, REBs overlapped genetic abnormalities as determined by aCGH. For example, both methods identified narrow regions of frequent amplification on chromosome 1p and narrow regions of frequent deletion on 17q. In a minority of cases, REBs were identified in regions not determined to be abnormal via other cytogenetic technologies. Specifically, expression biases reflective of cell proliferation were frequently identified on chromosome 6p21-23.

**Conclusion:**

Identification of REBs using a multiple span moving binomial test produced reasonable approximations of underlying cytogenetic abnormalities. However, caution should be used when attributing REBs identified on chromosome 6p to cytogenetic events in rapidly proliferating cells.

## Background

The parallel analysis of cytogenetic and transcriptional profiling data has revealed that changes in DNA copy number can have noticeable effects on gene expression. Studies comparing wild-type and mutant strains of yeast demonstrated that in regions of increased DNA copy number (i.e. genomic amplifications), the vast majority of genes that mapped within the amplified region had increased expression when compared to gene expression in non-amplified regions [1]. In this context, the unidirectional change in expression of a large number of adjacent genes can be termed a regional expression bias (REB). The dependence of gene expression on DNA copy number has also been observed with human derived samples, for example in a variety of aneuploid tumors and tumor derived cell lines, and in tissues obtained from patients with inherited trisomy disorders [2-13]. In these samples, ~40–70% of the genes that map to a cytogenetically abnormal region show corresponding expression changes; other genes within the region either do not change expression or, occasionally, change expression in the opposite direction of the cytogenetic abnormality. Nevertheless, as described in yeast, the majority of detectable regional gene expression biases in these mammalian tissues also coincide with chromosomal amplifications or deletions. As such, it is feasible to infer cytogenetic abnormalities by examination of high-density gene expression data. While the majority of REBs correspond to cytogenetic abnormalities, several groups have also identified a subset of regional gene expression biases that do not coincide with detectable DNA copy number changes [2,5,8,9,12,14]. While technical errors between DNA and expression-based approaches may account from some of these differences, it is also possible that other epigenetic factors could produce and regulate the appearance of REBs.

Partitioning gene expression data into subsets of adjacent genes and applying a summary function to each subset is a common method to identify REBs [1,5,11,15-19]. For example, a chromosome can be broken into consecutive, non-overlapping, 100 megabase (Mb) intervals and gene expression values that map to each interval tested for an expression biases using a variety of statistical/computational approaches. While partitioning approaches have been effective in identifying REBs, these approaches may be inherently limited due to the static nature of the partition span. Other, more dynamic, approaches to identify REBs utilizing run and scan statistics have also been reported [20]. However, the utility of these approaches for genome-wide scanning of expression biases is not well described.

Traditional data smoothing approaches ranging from simple moving averages to variable span local regression are common and straightforward methods that can be used to dampen variance and extract trends and patterns from ordered data series. For example, array comparative genomic hybridization (aCGH) data can be smoothed using an exponentially smoothed moving average to more easily identify abnormal chromosomal features [21]. While other approaches, such as hidden Markov models can also be utilized to analyze ordered genomic data [22], the complex nature of gene expression data may prevent the direct application of a subset of these types of analysis techniques. In this report, we outline an approach to identify REBs that summarizes the likelihood that each gene expression value measured lies within an regional expression bias using a multiple span moving binomial test. We use this approach to identify REBs in a set of hepatocellular carcinoma samples and compare the results to high resolution cytogenetic data obtained by aCGH. We also evaluated this approach using a set of clear cell renal cell carcinoma (ccRCC) gene expression profiles. In the majority of cases, dynamically determined REBs coincide with regions of DNA copy number change as determined by other molecular technologies. Interestingly, we identified a region on chromosome 6p where REBs are identified independent of apparent cytogenetic abnormalities. We show that the REBs in this region are produced in the most part by transcriptional responses to cellular proliferation.

## Results

### Identification of regional expression biases

**Figure 1:**
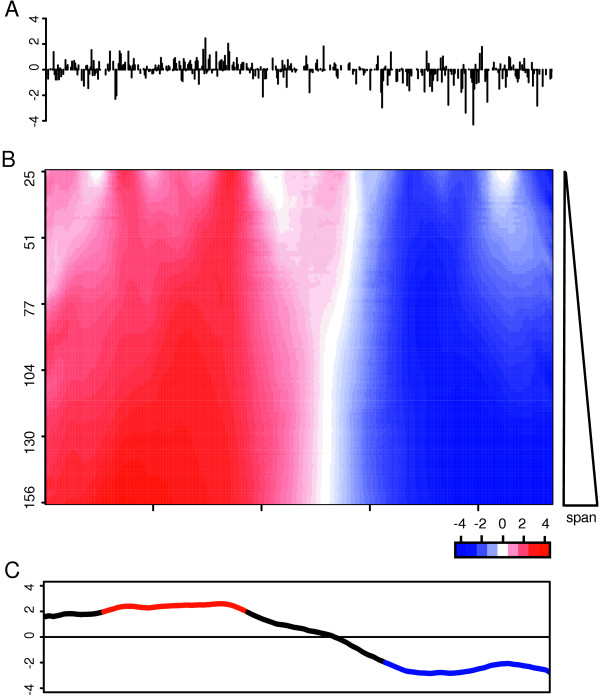
**Identification of regional expression biases**. A multiple span moving binomial test was applied to gene expression data to identify regional expression biases. **A**. Plot of log_2_-transformed tumor verses non-tumor expression ratios (*m *= 468) that map to chromosome 6 of sample HK1 organized from the p-arm telomere (left) to the q-arm telomere (right). **B**. Heatmap of the set of estimations generated by applying an approximated binomial function (see Materials and Methods) to the gene expression data using window spans of **i **= [25,...,*m*/3]. Genomic regions that contains a disproportionate number of relatively decreased expression values are shown in blue while genomic regions that show a disproportionate number of relatively increased expression values are shown in red. The color intensity indicates the significance of the expression bias. The highest intensity blue color indicates a *z*-score ≤ -4 while highest intensity red indicates a *z*-score ≥ 4 **C**. At each measured loci, an average *z*-score was computed from the set of estimations from each window span shown in **B **and plotted. Significantly down-regulated regional expression bias estimations are highlighted in blue (*z *≤ -1.96, p ≈ 0.05) and up-regulated bias estimations highlighted in red (*z *≥ 1.96, p ≈ 0.05).

To identify REBs, a modified version of a moving average is applied to two-color gene expression data obtained from the comparison of tumor HCC tissue to adjacent non-cancerous tissue (Figure 1a). Briefly, to calculate a moving average given a series of gene expression values ordered by genomic location and a window span that consists of five data points, the first five gene expression values would be collected, the average of this set determined, and the result stored as the first element of the moving average. The next span would include the second through the sixth gene expression values and the average of this span stored as the second element of the moving average. This process would continue until the end of the data series and the results of the moving average could be examined to identify trends. To identify REBs from ordered gene expression data, rather then a use an averaging function to evaluate each window span, an approximated binomial test is used to estimate of the probability, in terms of a *z*-score, that a gene expression bias exists within each span (see Materials and Methods). In this case, a positive *z*-score would indicate a disproportionate number of genes within the span show increased expression in the tumor profile when compared to the non-cancerous sample. Analogously, a negative *z*-score would indicate a disproportionate number of genes within the span show decreased expression in the tumor profile when compared to the non-cancerous samples. In addition, rather then collect data from a single window span, a data from a range of spans is collected and summarized (Figure 1b). In this case, the smallest window span used is 25, while the largest window size used is *n*/3 = 93. A minimum span of 25 assures the estimated *z*-scores are reasonably accurate (see Material and Methods) and a maximum span of *n*/3 prevents the generation of largely redundant data. Typical of many types of data smoothers, relatively small spans produce more variable REBs estimations while larger spans produce broader, more diffuse, REB estimations (Figure 1b). To estimate REB boundaries, for each gene loci the mean *z*-score derived from the range of window sizes is computed (Figure 1c). In addition, for plotting, the final REBs is masked so that only significant regions of bias are displayed. For simplicity, we term this approach IR-CGMA, for Improved Resolution-Comparative Genomic Microarray Analysis keeping in mind we have essentially described the application of an unweighted, multiple span, moving binomial test to identify REBs.

### Validation of IR-CGMA

**Figure 2:**
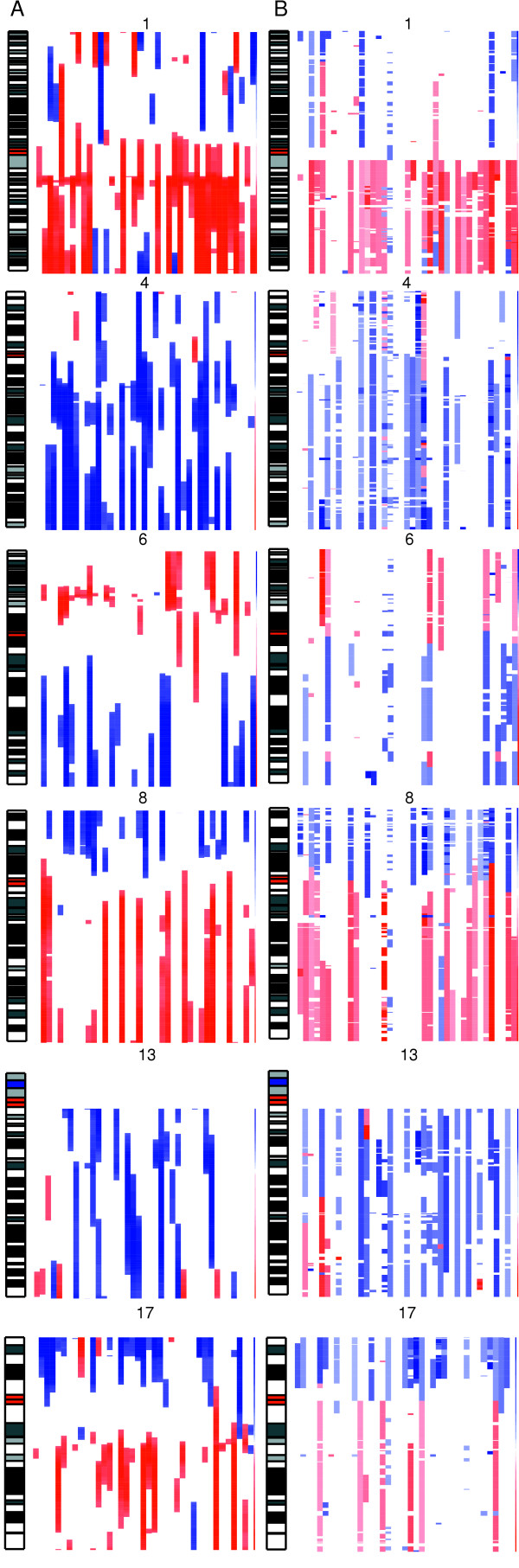
**Identification of REBs and DNA copy number abnormalities from individual HCC samples**. 39 HCC samples were analyzed for REBs from gene expression data using IR-CGMA and for DNA copy number abnormalities from aCGH data using hidden Markov modelling. Corresponding chromosome ideograms for chromosomes 1, 4, 6, 8, 13, and 17 are also shown to scale. The red bars in the ideogram highlight the centromere. **A**. IR-CGMA estimations were plotted as a heatmap to indicate significant expression biases as described in Figure 1. For consistent plotting, *z*-scores > 4 and *z*-scores < -4 were set to 4 and -4 respectively. Scales ranging from 4 to -4 are shown adjacent to each graph. Data for all autosomal chromosomes for all samples was also generated [see Additional file 1]. **B**. aCGH predictions of genomic deletions (*s *≤ -0.225, blue) and amplifications (*s *≥ 0.225, red). The highest intensity blue color indicates a s ≤ -1 while highest intensity red indicates s ≥ 1. Scales ranging from 1 to -1 are shown adjacent to each graph. Data for all autosomal chromosomes was also generated [see Additional file 2].

**Figure 3:**
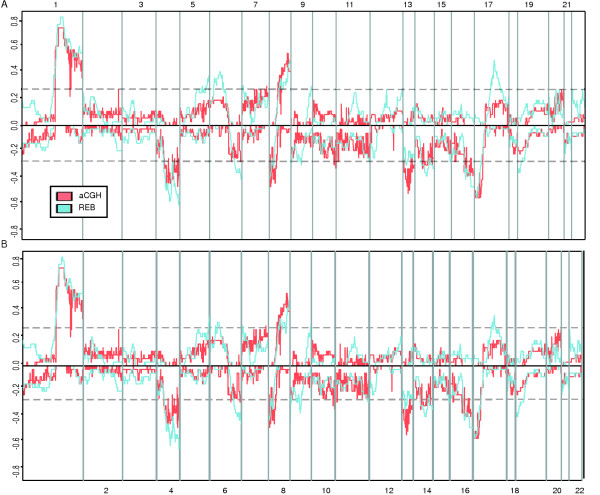
**Summary of REBs and DNA copy number changes in HCC**. A summary of the data generated as described in Figure 2. **A**. For each genetic loci on the autosomal chromosomes, the fraction of HCC samples that contained significant upwards expression biases were plotted as a positive fraction and the fraction of samples that contained significant downwards expression bias were plotted as a negative fraction. DNA copy number data determined by aCGH was plotted in a similar manner. **B**. Data was plotted as in **A**., with the exception that genes involved in cell proliferation and nucleic acid metabolism were removed, as described in the text, before REBs were identified.

To test the effectiveness of this method, we compared REBs identified by IR-CGMA to aCGH data derived from the same set of samples (Figure 2a, b). Both IR-CGMA and aCGH identified abnormalities that are commonly attributed to HCC such as +1q, -4q, -8p, +8q, -13q, -16q, -17p, and +17q [6]. To summarize the similarities and differences between IR.CGMA and aCGH, the predicted fractional allelic gain or loss was computed at each measured locus (Figure 3a). In the majority of cases, IR.CGMA identified frequent regional expression biases that corresponded to cytogenetic abnormalities as identified by aCGH. For example, on chromosome 1 both approaches identified a narrow region on the q-arm proximal to the centromere (1q21-23) that is frequently amplified. In addition, both approaches identified a region of frequent deletion on the distal tip of chromosome 17 (17p13). While in general REBs corresponded to features identified by aCGH there are regions of discrepancy. The most striking discrepancy between REBs and aCGH/CGH is located on chromosome 6p. Gain of chromosome 6p21-23 is not a frequently reported cytogenetic event in HCC either in this study or in other cytogenetic studies of HCC. However, chromosome 6p was frequently identified to be transcriptionally abnormal via REB scanning. Additionally, while gain of chromosome 17q frequently occurs in HCC, there is some discrepancy between the fraction of samples reported by IR-CGMA and aCGH.

**Figure 4:**
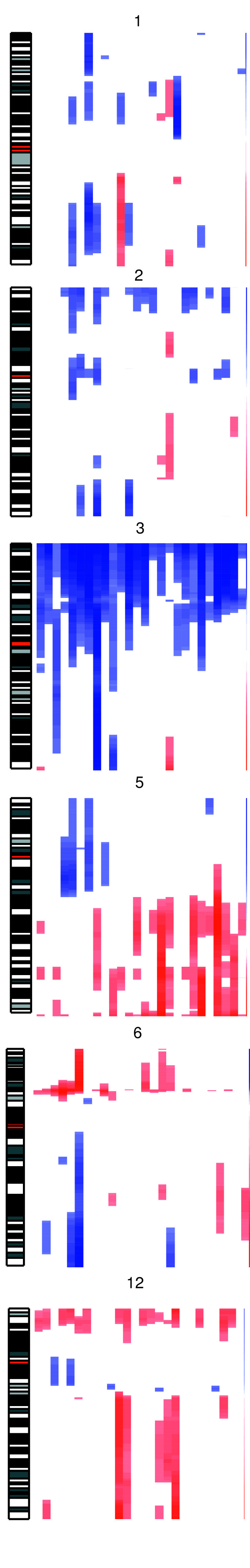
**Identification of REBs from individual ccRCC samples**. 27 ccRCC samples were analyzed for REBs and plotted as described in Figure 2a with the exception that chromosomes 1, 2, 3, 5, 6, and 12 are shown. Chromosomes 1 and 2 are shown as representative regions that do not frequent REBs. Data for all autosomal chromosomes was also generated [see Additional file 3].

**Figure 5:**
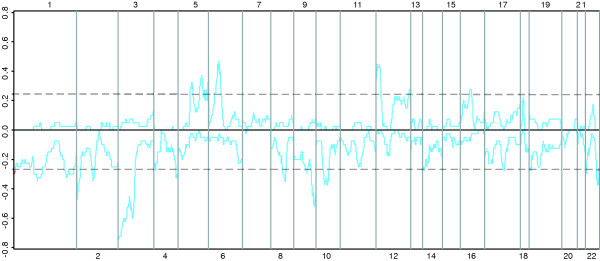
**Summary of REBs in ccRCC**. 27 ccRCC samples were analyzed for REBs and plotted as in Figure 3a.

Given these discrepancies, to provide additional validation for the use of a multiple span binomial test to identify regional expression biases, REBs were also identified from a set of gene expression data derived from clear cell renal cell carcinomas (ccRCC). Like HCC, ccRCC presents with a consistent set of cytogenetic abnormalities including loss of 3p and gain of 5p [23]. Frequent gain of chromosome 12p has also been reported in some CGH studies of ccRCC [24]. While in this study, we do not have corresponding cytogenetic data for these specific samples to perform direct comparisons, IR-CGMA did identify abnormalities that overlap genetic abnormalities frequently identified in ccRCC, including loss of 3p and gain of 5p (Figure 4, 5). Interestingly, gain of chromosome 6p is not a frequent cytogenetic abnormality associated with ccRCC, however, like the HCC samples, this region was frequently identified as being abnormal via REB scanning. While technical effects associated with either aCGH, traditional, CGH, or IR-CGMA may be responsible a subset of these discrepancies, it is also possible that epigenetic transcriptional regulation could contributes to the REBs. Therefore, to determine if the transcriptional abnormalities reflected certain types of epigenetic effects, we examined the gene expression data in more detail.

### Examination of chromosome 6p and 17q REBs

**Figure 6:**
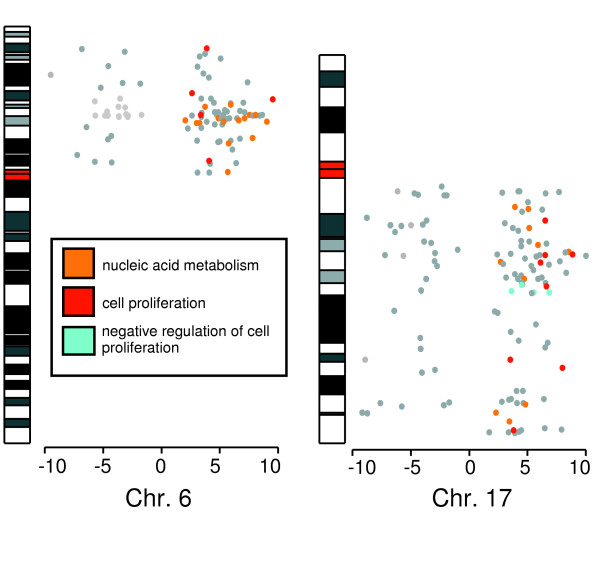
**Functional classification of differentially expressed genes on 6p and 17q**. Genes on chromosome 6p and chromosome 17q that are differentially expressed in HCC compared to adjacent non-cancerous tissue were identified as described in the Material and Methods. The *t*-statistic corresponding to each misregulated gene (p < 0.05) was plotted with respect to gene location. For consistent plotting, *t*-statistics > 10 and *t*-statistics < -10 were set to 10 and -10 respectively. Genes classified as nucleic acid metabolism, cell proliferation, and negative regulation of cell proliferation are highlighted orange, red, and cyan, respectively.

To evaluate the nature of the REBs on chromosomes 6p and 17q in HCC, misregulated genes within these regions were identified and partitioned based on Gene Ontology (Figure 6). Only two significantly enriched ontology's were identified (p < 0.005) from the upregulated genes in these regions: nucleic acid metabolism (GO:0006139) and cell proliferation (GO:0009607) [25,26]. While a small number of transcripts that had relatively increased expression in the tumor samples were identified as negative regulators of cell proliferation (GO:0008283), overall these results suggest that pronounced REBs on chromosome 6p and chromosome 17q reflect the active cell division of the tumor cells compared to non-cancerous cells. To test this hypothesis, up-regulated genes mapping to these ontologies were removed from the HCC gene expression dataset (154 of 8128 genes, 1.9%) and REBs recomputed (Figure 3b). The REBs on chromosome 6p were considerably diminished and the discrepancy on chromosome 17q was partially diminished. In contrast, REBs on chromosome 1q and 8q were not appreciably changed after removing the cell proliferation associated genes. Taken together, these results suggest that the transcriptional effects of active cell proliferation participate in the production of the REBs of 6p and 17q.

## Discussion

In this paper, we describe the construction and application of a straightforward data smoothing approach to identify REBs from gene expression data. As evidence for the validity of this approach, we demonstrate that REBs overlap cytogenetic abnormalities as determined using other cytogenetic profiling methods in the majority of cases. Due to the dependence of gene expression on chromosome dosage, identification of REBs can often assist in the interpretation of gene expression data. For example, detection of REBs can rapidly determine if a potential cytogenetic abnormality associates with particular sample classification, for example a more aggressive tumor subtype [27]. Perhaps more importantly, the prevalent overlap of transcriptional and cytogenetic abnormalities support HCC tumorigenesis models that advocate that recurrent cytogenetic aberrations, via their significant influences on gene expression, play important roles in HCC pathogenesis. In addition, the correlation between REBs and specific DNA copy number variation can assist in identification of candidate genes that have important function during tumorigenesis in a specific chromosomal regions. For example, a narrow region on the q-arm of chromosome 1 proximal to the centromere (1q21-23) is predicted to be frequently amplified both by IR.CGMA and aCGH, suggesting that this region may harbour candidate oncogenes. Inspection of genes that are highly expressed in 1q21-23 included several signalling molecules (MDUSP12, SHC1) and transcriptional factors (MEF2D, ILF2, TCFL1). Particular interesting is ephrin-A1, the ligand of Eph receptor tyrosine kinase. Ephrin-A1 has been implicated in angiogenesis and therefore may contribute to HCC development [28]. Clearly, it is important to evaluate the functions of these genes in HCC and determine the extent in which their gene expression is regulated by DNA amplification.

We also demonstrate in this study that not all REBs corresponded with detectable cytogenetic abnormalities, particularly in the region of chromosome 6p. Therefore, it is appropriate to apply alternative molecular approaches before attributing cytogenetic abnormalities to regional expression biases located in this region. Classification of the differentially expressed genes in this region into Gene Ontologies suggests that the regional expression changes reflect aspects of tumor cell proliferation as evidenced by an enrichment of features classified in nucleic acid metabolism and cell proliferation GO categories. Another notable feature of chromosome 6p, particularly 6p21-23, is that the gene density in this region is unusually high and harbors gene clusters of several protein families [29]. The unusually high gene density may also contribute to the identification of this region as frequently abnormal by REB scanning. It has been suggested that regions of high gene density correlates with open chromatin fibers. This open chromatin structure may facilitate transcriptional activation if appropriate transcriptional signals are present [30]. Other possible explanations of the REBs include regional methylation or Histone deacetylation.

While the high variability of gene expression data may prevent the direct application of several data modelling approaches, this study suggests that application of traditional data smoothing methods are appropriate to infer cytogenetic abnormalities from gene expression data and are worth investigating further. One potential disadvantage of smoothing approaches can be difficulty determining an appropriate window span that balances overall smoothness with optimal feature identification. While cross-validation using training and test data sets could theoretically identify an optimal window span for regional expression bias identification, we could not derive a span that was appropriate for all chromosomal regions across multiple data sets (data not shown). However, the increase in computer processing power allows the utilization of more computational intensive multiple span approaches to partially compensate for single span effects.

Unlike traditional cytogenetic analysis approaches, the resolution of this technique has a complex dependency on gene density, gene coverage on the array platform used, and tissue-dependent expression patterns. On average, the genome contains about 10 genes per Mb and varies between regions that have gene densities of ~6 genes per Mb (chromosome 13) to regions that have gene densities of ~26 genes per Mb (chromosome 19) [31]. As the smoothing approach presented requires at least 25 gene expression values to make a prediction, theoretically, the resolution of a REB could average ~2.5 Mb across the genome and range between ~1 to 4 Mb. However, for this analysis the cDNA arrays used contained ~8500 features could be confidently mapped to predicted genes. Of these features ~6000 genes (70%) where expressed at measurable levels in the liver tissue. Assuming ~30,000 human genes, the resolution for this study would be about 5-fold lower then the theoretically limits or average ~12.5 Mb across the genome and range from ~5 Mb to ~20 Mb.

While not reported here, this approach is suitable for single channel gene expression data provided appropriate reference and test expression profiling data can be converted to log-transformed expression ratios. We have also successfully used this approach to infer cytogenetic abnormalities from other species, such as mice and rats.

## Conclusion

In this report, we describe a method to identify regional expression biases using a multiple span moving binomial test. As evidence for the validity of this approach, we demonstrate that this methods identifies REBs that associate with cytogenetic abnormalities as determined by array CGH and traditional CGH in both hepatocellular carcinoma and clear cell renal cell carcinoma.

## Methods

### Pre-processing of gene expression data sets

Two-color gene expression profiles derived from 39 HCC tumor samples and corresponding non-cancerous liver samples [32], and 33 RCC and adjacent non-cancerous kidney tissue samples [33], were obtained from the Stanford Microarray Database [34]. In all cases, gene expression values were normalized using the within-print tip group normalization method as implemented in the BioConductor packages for the R environment [35,36]. Prior to normalization, R and G values were threshold such that R or G values <150 were set to 150. In these data sets, the cancerous and non-cancerous samples were compared to a pooled cell-line reference. To allow direct comparison of tumor to non-cancerous expression values, new gene expression ratios (R) were generated from tumor tissue ratio (*T/U*) and corresponding adjacent non-cancerous tissue ratios (*N/U*) such that R = log_2_(*T*/*U*) - log_2_(*N*/*U*) [2]. Sequence comparisons were used to map microarray probe sequences to predicted Ensembl transcripts (Ensembl version 19) [29]. Included in the Ensembl transcript annotations are chromosomal mapping locations at base-pair resolution. If multiple probes mapped to the same locus a mean gene expression value was utilized.

### Pre-processing of array comparative genomic hybridization data sets

Two-color array CGH data for the HCC samples was generated essentially as described [37]. A manuscript describing the details of the HCC copy number data and initial analysis is in preparation. In all cases, copy number values were transformed into copy number states using an unsupervised hidden Markov model as implemented in the BioConductor packages for the R environment [22,36]. States in which the median copy number change was ≥ 0.225 were defined as region of DNA gains and states in which the median copy number change ≤ -0.225 were defined as regions of DNA loss [37].

### Identification of regional expression biases (IR-CGMA method)

Gene expression values were separated into chromosome subsets and ordered by gene mapping location. A sliding window algorithm was applied to each ordered gene expression subset such that within each window span a binomial test was applied under the assumption that the probability (*p*) of the appearance of a positive relative gene expression value equals the probability (*q*) of the appearance of a negative relative expression value, *p *= *q *= 0.5, and a *z*-score for the span is computed using the normal approximation to the binomial distribution. The *z*-score can be converted to an approximate significance values based on the two-tailed *z*-statistic (*z*_a/2_) critical values. Data was generated using multiple window spans and an average *z*-score at each gene location was computed. More formally, given a set of ordered gene expression values *g*_*j *_for genes *j *= 1, 2, ...*m*, let *x*_*ij *_denote expression bias approximations for genes *j *= 1, 2, ...*m *using window spans *i *= 25, 26, 27, ...*m*/3 where *n *denotes the number of window spans examined. An empty matrix **X**_[n#m] _is populated such that  for *m*-*i*+2 >*j *≥ *i *where *t *denotes the number of non-zero and *r *the number of positive values within the span {*g*_*k*_, *g*_*k*+1_, ...*g*_*k*+*i*-1_}. To not discard regions, *x*_*ij *_is tapered when *j *<*i *such that  and analogously tapered when *j *≥ *m*-*i*+2. Final regional expression bias estimates (*b*_*j*_) are computed such that . Performing IR-CGMA on the 39 HCC gene expression profiles took approximately five minutes on a 2.6 GHz Intel Pentium IV with 1 GB of RAM.

### Identification of misregulated genes

Identification of misregulated genes from the HCC gene expression profiles occurred in two-steps. First, genes were filtered to ensure each gene was well measured across the data set using an exact binomial test (p < 0.05). In this case, data was required in 24 of 39 (64%) of samples. Next, a one-sample t-test assuming unequal variance was applied to determine if expression values were significantly misregulated (p < 0.05).

## Authors' contributions

KF and KD designed and implemented the data analysis algorithms and performed the data analysis. XC and CH obtained the HCC samples and generated both the gene expression and array CGH data. KF and XC collaborated to write the manuscript. All authors read and approved the final manuscript.

## Supplementary Material

Additional File 1 Regional expression biases for all chromosomes in the HCC samples Click here for file

Additional File 2 aCGH states for all chromosomes in the HCC samples Click here for file

Additional File 3 Regional expression biases for all chromosomes in the RCC samples Click here for file
