# Supplementary figures and images for: Comparison of array-based comparative genomic hybridization with gene expression-based regional expression biases to identify genetic abnormalities in hepatocellular carcinoma
Source: BMC Genomics. 2005 May 9;6:67. doi: 10.1186/1471-2164-6-67 (PMC1134655; doi:10.1186/1471-2164-6-67)

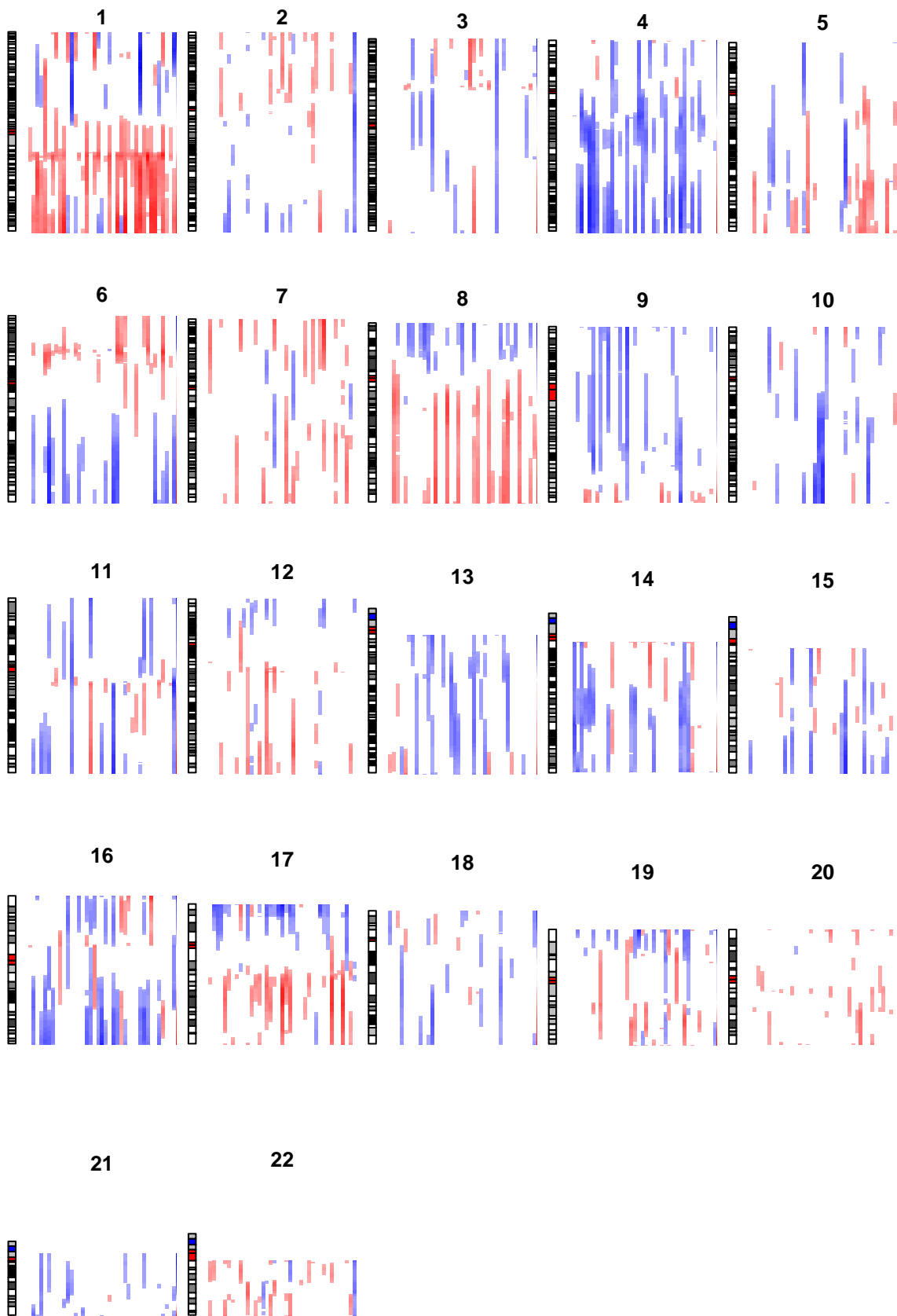

Supplement: Additional File 1 — Regional expression biases for all chromosomes in the HCC samples [file 1471-2164-6-67-S1.pdf]

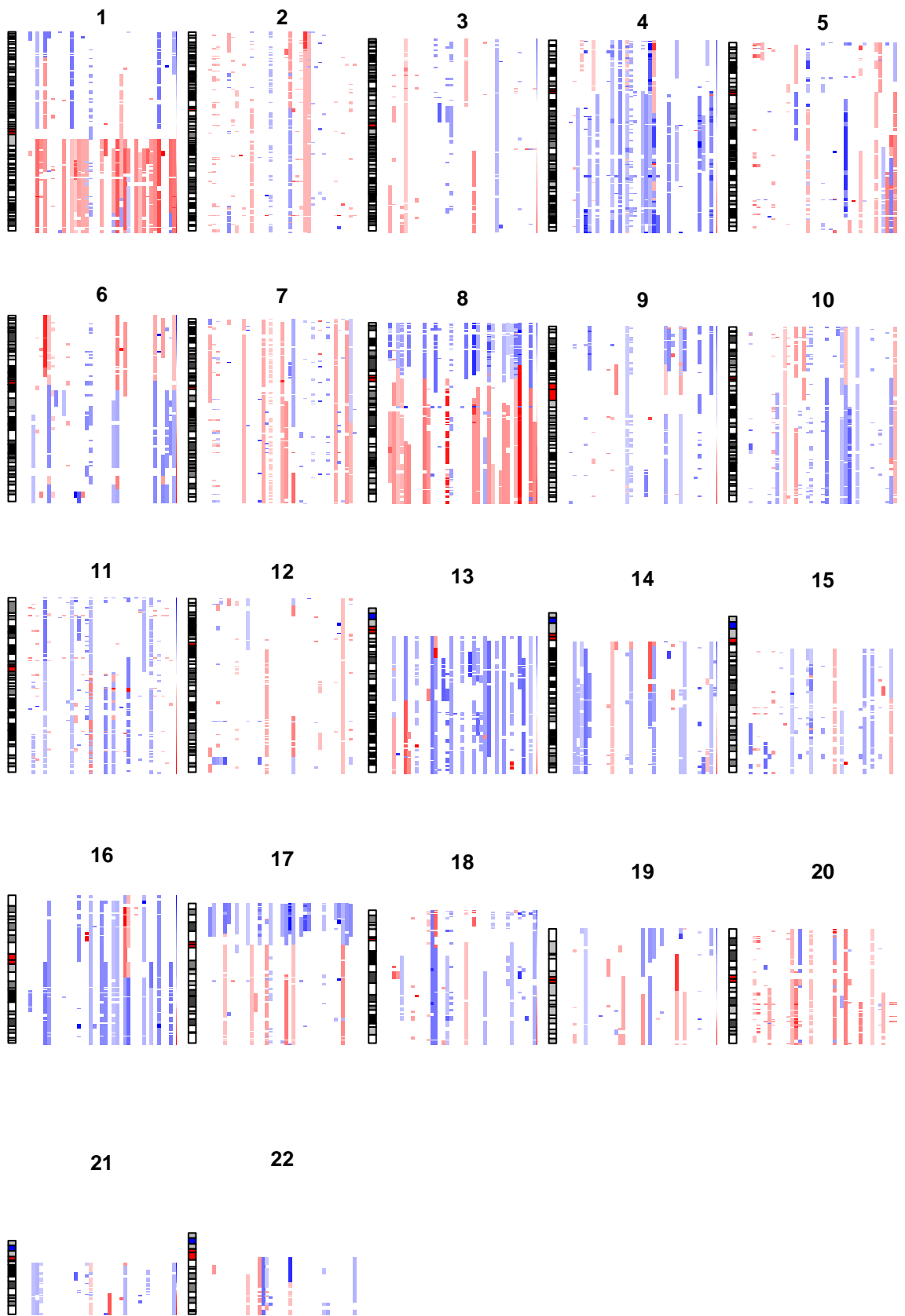

Supplement: Additional File 2 — aCGH states for all chromosomes in the HCC samples [file 1471-2164-6-67-S2.pdf]

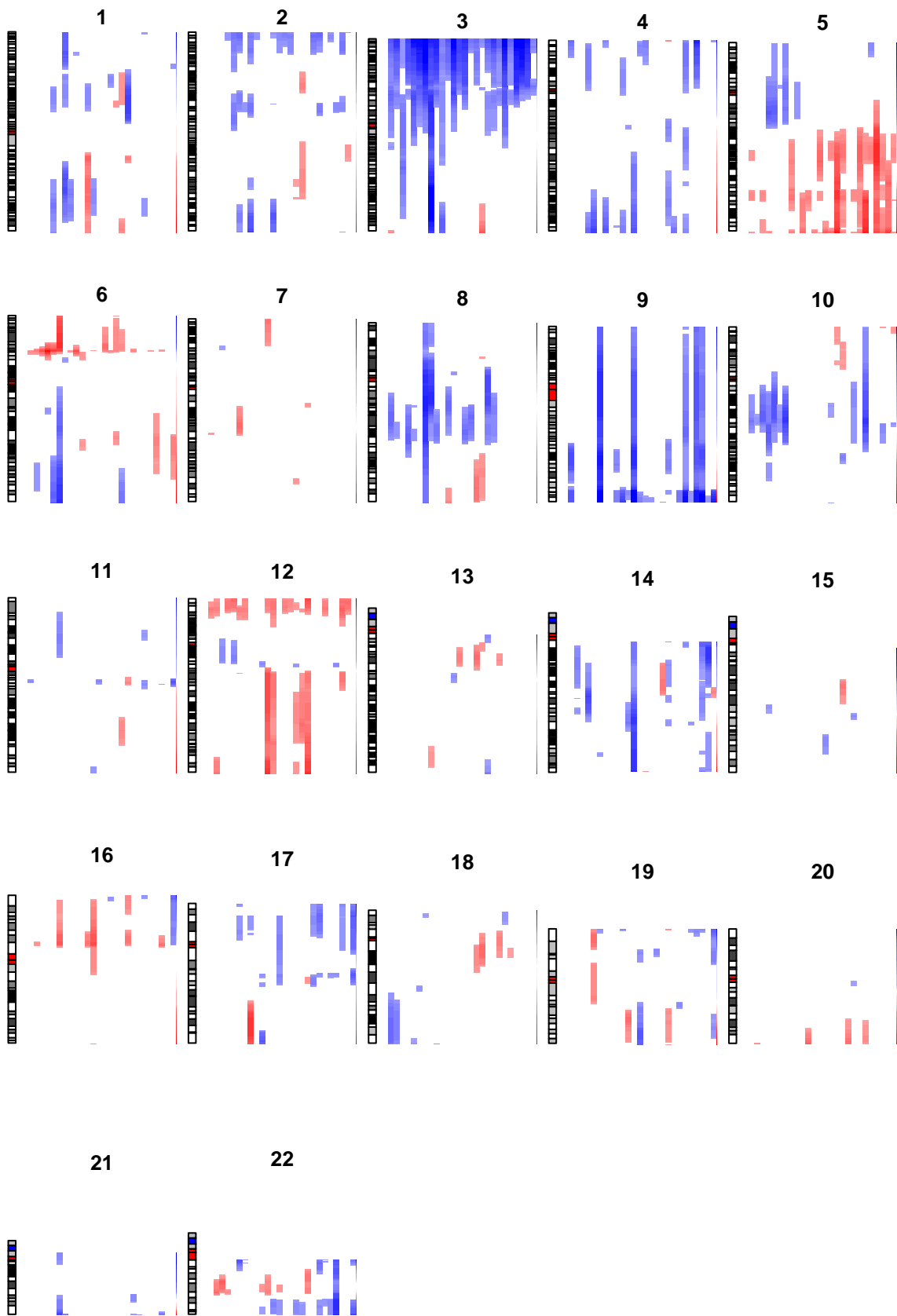

Supplement: Additional File 3 — Regional expression biases for all chromosomes in the RCC samples [file 1471-2164-6-67-S3.pdf]
